# Genetically Modified Flax Expressing NAP-SsGT1 Transgene: Examination of Anti-Inflammatory Action

**DOI:** 10.3390/ijms150916741

**Published:** 2014-09-22

**Authors:** Magdalena Matusiewicz, Iwona Kosieradzka, Magdalena Zuk, Jan Szopa

**Affiliations:** 1 Department of Animal Nutrition and Biotechnology, Faculty of Animal Sciences, Warsaw University of Life Sciences, Ciszewskiego 8, 02-786 Warsaw, Poland; E-Mail: iwona_kosieradzka@sggw.pl; 2 Department of Genetic Biochemistry, Faculty of Biotechnology, University of Wrocław, Przybyszewskiego 63/77, 51-148 Wrocław, Poland; E-Mails: mzuk@ibmb.uni.wroc.pl (M.Z.); szopa@ibmb.uni.wroc.pl (J.S.)

**Keywords:** genetically modified flax, seed cake, flavonoid glycosides, inflammation, food safety, mouse

## Abstract

The aim of the work was to define the influence of dietary supplementation with GM (genetically modified) GT#4 flaxseed cake enriched in polyphenols on inflammation development in mice liver. Mice were given *ad libitum* isoprotein diets: (1) standard diet; (2) high-fat diet rich in lard, high-fat diet enriched with 30% of (3) isogenic flax Linola seed cake; and (4) GM GT#4 flaxseed cake; for 96 days. Administration of transgenic and isogenic seed cake lowered body weight gain, of transgenic to the standard diet level. Serum total antioxidant status was statistically significantly improved in GT#4 flaxseed cake group and did not differ from Linola. Serum thiobarbituric acid reactive substances, lipid profile and the liver concentration of pro-inflammatory cytokine tumor necrosis factor-α were ameliorated by GM and isogenic flaxseed cake consumption. The level of pro-inflammatory cytokine interferon-γ did not differ between mice obtaining GM GT#4 and non-GM flaxseed cakes. The C-reactive protein concentration was reduced in animals fed GT#4 flaxseed cake and did not differ from those fed non-GM flaxseed cake-based diet. Similarly, the liver structure of mice consuming diets enriched in flaxseed cake was improved. Dietetic enrichment with GM GT#4 and non-GM flaxseed cakes may be a promising solution for health problems resulting from improper diet.

## 1. Introduction

Obesity, a positive balance of energy as a result of excessive consumption, its insufficient expenditure, or both [1], is growing at a dangerous rate and is becoming a major concern of public health [2]. Recent studies have increasingly highlighted the association of obesity with inflammation [3] that is chronic, total and has deceitful results [4,5,6,7,8]. Crucial stress pathways connected with obesity could be activated by reactive oxygen species (ROS), cytokines and other factors produced by the cells of the immune system and adipocytes, possible disturbing important metabolic processes [3].

The potential for natural products to prevent obesity is an area of considerable interest for research in a food field [9,10,11]. Plant foods contain polyphenols, a significant family of phytochemicals with health benefits [12] that may constitute an important element of functional diet for people with metabolic function disorders. Good source of polyphenols and other substances that have potentially beneficial effects for organism are flaxseeds (*Linum usitatissimum*). Numerous preclinical studies have shown that polyphenols exhibit protective actions on many pathologies caused by oxidative stress, as disorders of metabolism, they may suppress adipose tissue growth [13,14]. Flavonoids, a class of polyphenols, form a grand family of 6000 phytochemicals present in fruits and vegetables. Many studies concentrate on their antioxidant [15] and anti-inflammatory [16] actions. Although flavonoids are considered to be agents with non-nutritive properties, they are gaining interest as a result of their potential function in major chronic disease prevention [17,18,19,20].

One of the major flavonoids determining plant dietetic value is quercetin. This flavonoid is not present as aglycon (without sugar groups) in food, but is differently glycosylated. Its bioavailability depends on glycoside types present in food sources. The bioavailability of quercetin glycosides present in different food is different [21]. Absorption of quercetin glycosides almost doubles the corresponding aglycon [22]. After absorption, it is metabolized in small intestines, colon, liver and kidneys [23]. As a result of absorption and metabolism, quercetin from the diet is present at the nanomolar range in plasma [21,24]. The half-lives of quercetin and its metabolites is between 11 and 28 h, there is a possibility of significant elevation of plasma levels upon continuous supplementation [21,25]. One of the polyphenol classes—phenolic acids—show a full range of pharmacological properties including antioxidant, hypolipidemic, anti-inflammatory and anticancer [17,26,27,28]. A second class of polyphenols—lignans—a phytoestrogen group where flaxseeds are the richest dietary source, contain secoisolariciresinol (>3.7 g/kg dry weight) and small quantities of matairesinol [29]. During consumption, secoisolariciresinol and matairesinol are converted to enterodiol and enterolactone, the mammalian lignans with several biological activities, including antioxidative and estrogen-like, which may lower chronic disease risk including hormone-based obesity [29,30].

Flax is grown for commercial use in over 30 countries of the world. Flaxseed oil has long been used in human and animal diet. This oil is one of the richest sources of human linolenic and linoleic polyunsaturated fatty acids (PUFA) that must be ingested with food since they cannot be synthesized in the organism. Lipids in flax grains could be protected against oxidation by the antioxidative effect of polyphenols. These hydrophilic compounds even after cold extraction remain associated with seed cake. Overproduction of various natural antioxidants in flax grain could be achieved by the genetic engineering approach. Overexpression of glycosyltransferase from *Solanum sogarandinum* (SsGT1) resulted in a higher quantity of flavonoid glycosides, and increases stability as well as proanthocyanin, lignan, phenolic acid and unsaturated fatty acid accumulation in the seeds [31].

These investigations have engendered interest in development of a nutritional supplement that would allow for high and convenient dose of polyphenol administration, which could be useful for treatment or prevention of inflammatory state related to diet-induced obesity.

The aim of this study was to examine the influence of the chronic administration of cakes prepared with GM GT#4 flaxseeds, overexpressing glycosyltransferase, on the disturbances induced by high-fat diet consumption in mice including changes in the red-ox state, lipid profile, inflammatory state of the liver and their (ultra)structure. In order to obtain the seed cakes, flaxseeds were grounded and transferred to industrial worm gear oil press (Oil Press DD85G—IBG Monoforts Oekotec GmbH & Co., Mönchengladbach, Germany) toward cold pressing of oil. The mean yield of procedure was 75% of seed cake and 25% of oil. The dose of GM (genetically modified) GT#4 and non-GM flaxseed cakes were established to not cause nutritional imbalance or metabolic disturbances in the tested animals [32,33]. On a basis of compositional analysis, preliminary studies and review of literature, tested dose of seed cake did not contain inherent and anti-nutritional components in an unsafely concentration for organism (isogenic Linola flaxseeds contained 189.33 mg of linustatin/100 g fresh weight and 103.5 mg of neolinustatin/100 g fresh weight, GM flaxseeds comprised 164.17 mg of linustatin/100 g fresh weight and 93.50 of neolinustatin/100 g fresh weight, gas chromatography-mass spectrometry method, own unpublished data). We used mice, a widely used animal model, that were administered *ad libitum* the isoprotein diets: standard diet (SD), high-fat diet (HFD) with pork lard, and diets supplemented with 30% of seed cake of non-GM flax Linola (Linola) or GM flax GT#4 (GT#4). On the 96th day, after an overnight fasting, animals were anaesthetized, and blood and livers were collected for the analysis. The food intake, body weight gain, red-ox homeostasis indicators (total antioxidant status, TAS; thiobarbituric acid reactive substances, TBARS) and lipid profile in serum were determined. Inflammation markers (tumor necrosis factor-α, TNF-α; interferon-γ, IFN-γ; interleukin-6, IL-6; adiponectin, ADP; C-reactive protein, CRP) were measured in liver and changes in its (ultra)structure were assessed.

## 2. Results and Discussion

### 2.1. Effects of Feeding with GM (Genetically Modified) GT#4 Flaxseed Cake on Body Weight Gain of Mice and Food Intake

**Figure 1:**
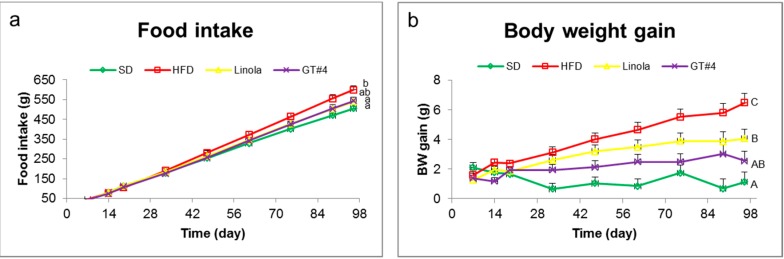
(**a**) Food intake of mice fed different diets; (**b**) Body weight gain. The error bars indicate the standard error of the mean (SEM). Significant effect: values that do not share a common small letter (a,b) differ significantly at the level *p* < 0.05, and values that do not share a common capital letter (A,B,C) differ significantly at the level *p* < 0.01.

There were no statistically significant differences in food intake of a diet supplemented with 30% of GM line GT#4 and non-GM control flaxseed cakes from (Figure 1a). The intake of a diet with GT#4 flaxseed cake was the same as SD and HFD. The statistically significant differences in body weight gain of mice occurred in the 14th day of a study (Figure 1b). After the feeding for 96 days, there were no statistically significant differences in body weight gain of mice that administered diets supplemented with a 30% of GM GT#4 and non-GM flaxseed cakes. Body weight gain of mice fed these diets was significantly lower than that of the animals which consumed HFD. There were no differences in body weight gain in animals under GM GT#4 flax and SD diets. These results clearly suggest that diet supplementation with GM GT#4 and isogenic flaxseed cakes may decrease the development of obesity.

Taking into consideration studies in obese humans and rodents the influence of quercetin on body weight was not clear. In obese male Zucker rats, only the higher dose of quercetin (10 mg/kg BW/day) but not the lower one (2 mg/kg BW/day), were administered for 10 weeks, to reduce body weight gain [34]. Furthermore, a reduction in body weight gain was noted only at high concentrations of quercetin (0.2% and 0.5% in a diet) in the male Sprague-Dawley rats fed high-fat high-sucrose (HFHS) diet for 4 weeks [35]. In male C57BL/6J mice fed the HFD for 8 weeks, supplementation with high concentration of quercetin (0.8%) did not influence body weight [36]. Feeding the C57BL/6J female mice SD enriched with quercetin (0.1 mg/g diet) over 6 weeks did not influence body weight gain [37]. Additionally, overweight-obese humans receiving 150 mg/day of quercetin for 6 weeks experienced no significant changes in body weight, circumference of waist, fat and fat-free mass [38]. Dietary supplementation of ferulic acid (0.5%) for 7 weeks suppressed body weight gain of C57BL/6J male mice fed the HFD [39]. Addition of ferulic acid (0.5%) to hypercholesterolemic diet did not influence the body weight of Golden Syrian hamsters during a 10-week period [40].

Concentration of single polyphenols in flaxseed cakes [31,41], that constitute the experimental material in our study, was lower compared to concentrations of synthetic ones used in presented experiments. That may explain the lack of significant differences in body weight gain observed between GM and non-GM flaxseed cakes-based diets.

### 2.2. Effects of Administration of GM GT#4 Flaxseed Cake on Red-ox State Indices in Mice Serum

**Figure 2:**
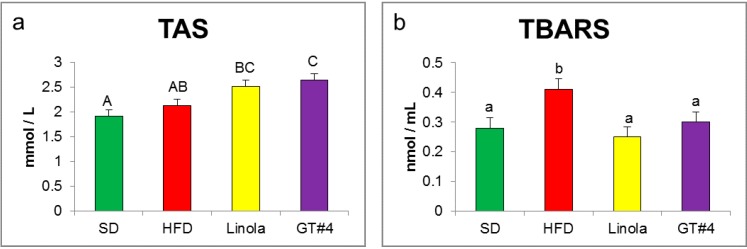
The effect of the experimental diet consumption on the red-ox state indices. (**a**) TAS (total antioxidant status); (**b**) TBARS (thiobarbituric acid reactive substances). The error bars indicate the SEM. Significant effect: values that do not share a common small letter (a,b) differ significantly at the level *p* < 0.05, and values that do not share a common capital letter (A,B,C) differ significantly at the level *p* < 0.01.

In the present study, the TAS (Figure 2a), indicating total level of antioxidants defending against pro-oxidative, damaging activity of free radicals and ROS, in serum of mice fed a diet supplemented with GM GT#4 flaxseed cake was significantly higher comparing to SD and HFD groups, and did not differ from Linola group (*p* < 0.01). A product of lipid peroxidation, malondialdehyde (MDA), was detected to evaluate the oxidative stress degree [42]. MDA can trigger mutagenic lesions in DNA that could be involved in various diseases pathology [43]. As was shown in Figure 2b, in serum of mice fed the diets with addition of GT#4 and Linola seed cake significant decrease in a content of TBARS expressed as MDA was observed comparing to HFD (*p* < 0.05). There were no differences in MDA level between flax groups and SD group (*p* < 0.05).

Arts *et al.* [44] have documented that quercetin (one of the most important flavonols whose concentration was increased in GM GT#4 flaxseed cake) total antioxidant capacity in plasma is more than six times higher than trolox, the reference antioxidant. Secoisolariciresinol diglucoside (SDG) concentration of 15 mg/kg BW/d, a plant lignan derived from flaxseeds, enriched in the GM GT#4 flax line [31], has been shown to reduce oxidative stress. It diminished aortic lipid peroxidation and increased antioxidant reserve in New Zealand white rabbits fed 1% cholesterol diet in a 8-week study that could be explained by its antioxidant activity [45]. Level of TBARS in plasma and erythrocytes were reduced in mice fed HFD supplemented with ferulic acid, the major phenolic acid whose concentration was elevated in GM line GT#4 flaxseed cake, and could inhibit oxidative stress induced by HFD by scavenging free radicals [39]. Supplementation with flaxseeds (40 g per day) for 12 weeks lowered plasma TBARS concentration in obese glucose intolerant people [46].

### 2.3. Influence of Consumption of GM Flax GT#4 Seed Cake on Mice Serum Lipid Profile

**Figure 3:**
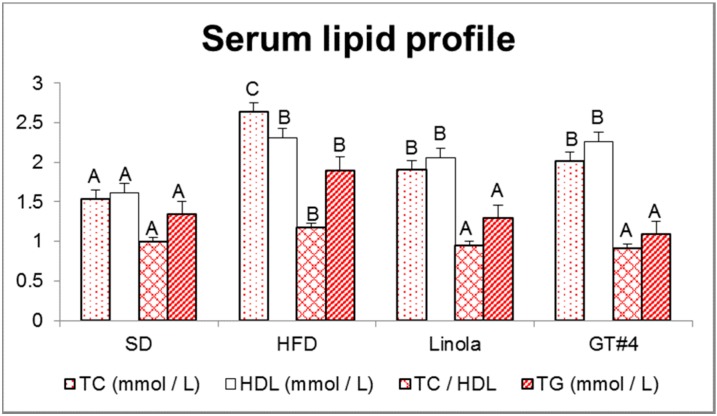
Lipid profile of serum of animals fed the experimental diets. The error bars indicate the SEM. Significant effect: values that do not share a common letter (A,B,C) differ significantly (*p* < 0.01).

Lipid profile of serum was given in Figure 3. The concentration of total cholesterol (TC) was significantly decreased in animals that administered diet supplemented with GM GT#4 and Linola flaxseed cakes comparing to HFD. High-density lipoprotein (HDL) cholesterol level was markedly elevated in groups containing both types of flaxseed cakes and HFD. Moreover, TC/HDL ratio and triglycerides (TG) concentration were decreased after the feeding of GM flax and their isogenic control in comparison with HFD, and both flax groups did not differ significantly from SD. These results suggest that administration of seed cake of GM GT#4 and non-GM flaxseed cakes had lipid-lowering effect.

It has been demonstrated that quercetin has profitable influence on the lipid profile. Quercetin glucoside (0.1%) reduced plasma TC and TG in New Zealand white rabbits fed high-cholesterol diet for one month [47]. The hypolipidemic effect could be due to cholesterol absorption inhibition in the intestine and cholesterol synthesis suppression in the liver by the quercetin glucoside, resulting in lower lipoprotein secretion to plasma and a decrease in TC level. The lower level of TG could be due to lipid metabolism affected by the quercetin glucoside elevation of the enzyme activity of β-oxidation. Ferulic acid lowered the plasma concentration of TC and TG, and elevated HDL in HFD-fed mice [39]. It appears that ferulic acid improves plasma lipid profile by increasing faecal lipid excretion. Addition of ferulic acid to hypercholesterolemic diet lowered plasma TC, non-HDL cholesterol, TC/HDL and HDL, and did not affect TG concentration in hamsters [40]. The cholesterol-lowering mechanism was unexplained at that time. SDG derived from flaxseed lowered serum TC, low-density lipoprotein (LDL) cholesterol and increased HDL in hypercholesterolemic rabbits [45]. This effect could be due to metabolites of SDG (secoisolariciresinol, enterodiol and enterolactone) antioxidant activity, up to five times higher than Vitamin E [30]. In human studies, flaxseeds could reduce TC and LDL, without affecting HDL and TG levels [48].

In this investigation, the lipid profile improvement was associated with an administration of flaxseed cakes in a high-fat diet and it was independent from genetic modification.

### 2.4. Effects of GM GT#4 Flaxseed Cake Consumption on Inflammatory State Biomarkers in Mice Liver

**Figure 4:**
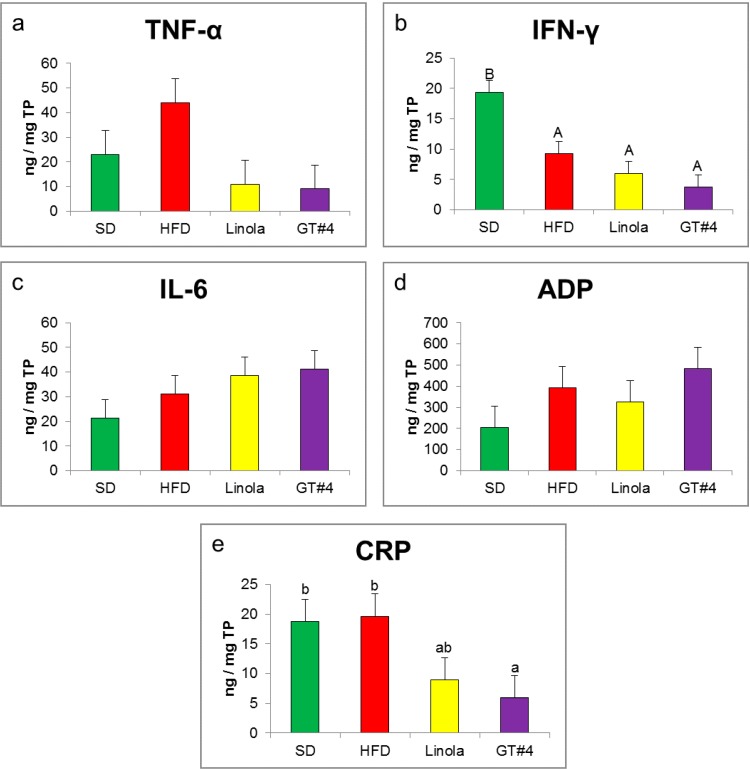
The influence of feeding with experimental diets on the level of pro- and anti-inflammatory state markers in mice liver. (**a**) TNF-α (**b**) IFN-γ (**c**) IL-6 (**d**) ADP and (**e**) CRP. The error bars indicate the SEM. Significant effect: values that do not share a common small letter (a,b) differ significantly at the level *p* < 0.05, and values that do not share a common capital letter (A,B) differ significantly at the level *p* < 0.01.

The level of pro-inflammatory cytokine TNF-α in the liver of mice fed the diet containing GM GT#4 flaxseed cake did not differ significantly from animals that were administered non-GM flaxseed cake (Figure 4a). These values were lower compared to those obtained in the HFD group. The concentration of second pro-inflammatory cytokine IFN-γ in the liver of mice that consumed GT#4 flaxseed cake did not differ from Linola flaxseed cake-based diet (*p* < 0.01) (Figure 4b). There were no differences in the concentration of pro-inflammatory cytokine IL-6 and anti-inflammatory adipocytokine ADP (Figure 4c,d). The level of CRP—an acute phase protein of which an increase could indicate chronic inflammation—did not differ between mice fed GT#4 flax and non-GM seed cakes (*p* < 0.05). The concentration of this protein was smaller in mice fed GT#4 flaxseed cake compared to HFD-fed (*p* < 0.05) (Figure 4e).

Previous studies have shown that liver inflammatory pathways are to a great extent regulated by the cytokines. TNF-dependent signalization in liver is crucial for controlling cell homeostasis [49]. A positive correlation has been found between TNF-α plasma level and body mass index (BMI) [50]. Serum and intrahepatic IL-6 concentrations are increased in patients that have acute or chronic liver diseases [49]. Production of ADP is diminished with increased adiposity [50,51,52] and its low level is associated with Metabolic Syndrome [53], being a consequence of overnutrition, the consumption of diet high in energy. CRP, a biomarker of chronic inflammatory state, has been shown to be positively associated with BMI. CRP production in liver could be regulated by TNF-α and IL-6 [50]. Some *in vitro* studies with different lines of cells have shown that the main flavonoid overproduced in GT#4 flaxseed cake, quercetin, could inhibit cytokine production induced by the bacterial endotoxin lipopolysaccharide (LPS); for instance. production of TNF-α in macrophages [54]. Quercetin and its major metabolite isorhamnetin lowered TNF-α and IL-6 mRNA levels and TNF-α secretion in murine macrophages stimulated with LPS. Plasma TNF-α concentrations were lowered in mice fed the SD enriched with quercetin compared to controls [37]. Quercetin reduced inflammation in isolated macrophages and adipocytes of human [55]. Macrophages treated with quercetin had diminished expression of TNF-α and IL-6, and this flavonoid decreased macrophage-mediated induction of TNF-α expression in human adipocytes. This flavonoid attenuated induced by TNF-α expression and secretion of IL-6 in human adipocytes [56]. Quercetin could inhibit the production and gene expression of TNF-α in peripheral blood mononuclear cells via NF-κB modulation [57]. A possible anti-inflammatory action of quercetin may be explained by the oxidative stress and inflammation interplay. ROS are involved in the promotion of inflammatory processes via activation of transcription factors such as NF-κB which induce cytokine production, like TNF-α [58]. Production of TNF-α by the visceral adipose tissue was elevated in obese rats compared to lean [34]. The daily administration of higher dose of the main flavonol overexpressed in seed cake of GM GT#4 flax, quercetin (10 mg/kg BW), reduced TNF-α production in obese rats, but lower quercetin dose (2 mg/kg BW) did not produce this effect. The plasma concentration of ADP was lower in obese rats than lean. A daily intake of higher quercetin dose increased concentration of ADP in obese rats. Only a higher dose of quercetin seems to result in anti-inflammatory action. Dietary quercetin reduced circulating plasma IFN-γ in mice fed HFD [36]. This and other results suggest potential use of quercetin in associated with obesity chronic low-level inflammation. Genetically modified flavonoid-enriched tomato intake (4 g/kg of GM tomato peel) lowered CRP—in human CRP transgenic mice overexpressing indices of cardiovascular risk—more than non-GM tomato [59]. Although it had been shown that plasma CRP concentration is not related to intake of flavonoids [60], some data demonstrate that flavonoids could diminish CRP at the protein level in liver cells and this effect depends on dose [61]. There were no differences in the inflammatory state markers’ (CRP, ADP, IL-6) concentrations in overweight and obese men and postmenopausal women in the pre-diabetes state consuming flaxseeds (13 or 26 g/day) for 12 weeks [62]. Daily consumption (40 g) of flaxseeds by healthy menopausal women for 12 months did not influence the concentrations of CRP [63]. Flaxseed supplementation (40 g/day) for 12 weeks did not affect plasma CRP, TNF-α and IL-6 levels in obese glucose intolerant people. These parameters were in normal ranges, and the reason could be that in obese participants of the experiment did not identify the low grade systemic inflammatory state [46].

### 2.5. Effects of Administration of GM GT#4 Flaxseed Cake on Liver Histological Changes in Mice

**Figure 5:**
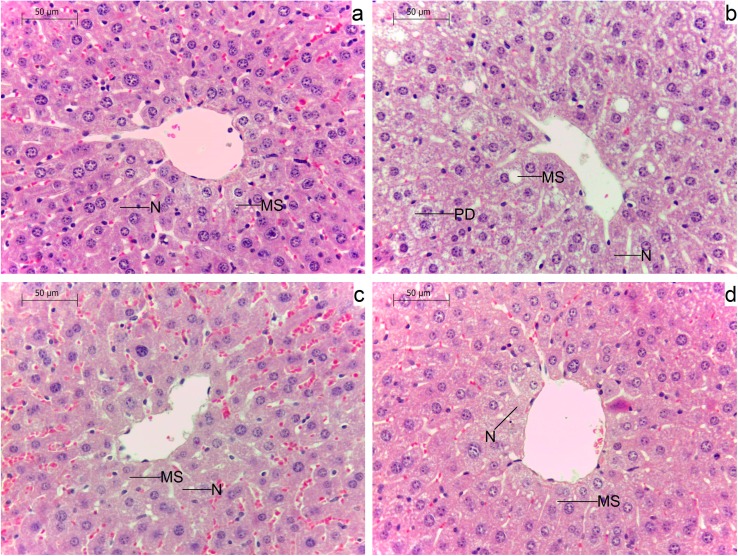
Histopathological examination of mice liver by hematoxylin and eosin staining. Animals administered different diets: (**a**) SD (standard diet); (**b**) HFD (high-fat diet); (**c**) Linola; and (**d**) GT#4. Parenchymatous degeneration (PD), microvesicular steatosis (MS), necrosis (N). 20× magnification.

Hematoxylin and eosin (H&E) stained liver sections are shown in Figure 5. Histological examination of liver of mice fed HFD (Figure 5b) demonstrated parenchymatous degeneration of hepatocytes. In hepatocytes of animals that were administered this diet, microvesicular steatosis (numerous small fat vacuoles that do not displace centrally located nucleus) was also presented. Additionally, disseminated necrosis of hepatocytes was affirmed. The structure of the liver of animals that consumed diet with addition of GM GT#4 and non-GM flaxseed cakes was similar to mice fed SD (Figure 5a,c,d). In those groups, less lipid vacuoles were observed in hepatocyte cytoplasm and only single cells underwent necrosis.

HFHS diets could contribute to non-alcoholic fatty liver disease induction. It has been reported that some polyphenols reduce lipid accumulation. The liver histological feature of male Wistar rats fed HFHS diet for 6 weeks was hepatic macrosteatosis (large droplets in which single large lipid vacuole distends the cell pushing to the side the nucleus and cytoplasm). Supplementation of this diet with a polyphenol extract from red wine (2 g/kg diet) resulted in a microvesicular steatosis. Therefore, polyphenol administration partially prevented hepatic steatosis induced by the HFHS diet [64]. Female Wistar rats fed a 1% cholesterol diet supplemented with SDG (3 and 6 mg/kg BW/day) and its aglycone metabolite secoisolariciresinol (3.2 mg/kg BW/day) for 4 weeks exhibited decreased hepatic lipidosis that correlated with serum lipid parameters [65]. Administration of flaxseed oil rich in α-linolenic acid, for 60 days, protected the liver of HFD-fed male Wistar rats and reduced the hepatic lipids suggesting participation in regulation of lipid metabolism [66]. Supplementation of HFD with quercetin (100 mg/rat/day) did not influence liver fat of male Wistar rats during a 10-week study [67]. Daily addition of quercetin (30–60 mg/kg BW) for 14 days exhibited hepatoprotective effects in nonalcoholic steatohepatitis induced in male gerbils HFD-fed for 28 days. Lipid accumulation could be decreased in the hepatocytes of quercetin groups which was in agreement with serum lipid profiles. Quercetin treatment also reduced liver fibrosis and lobular inflammation comparing with animals fed HFD [68]. Quercetin supplementation (0.025%) reduced hepatic lipid accumulation induced by HFD in male C57BL/6J mice in a 9-week experiment probably due to regulation of expression of genes related to lipid metabolism, and therefore decreased hepatic fatty acids and triglycerides biosynthesis [69]. Histological assessment of liver tissue indicated a beneficial influence of transgenic and isogenic flaxseed cakes as components of high-fat diets.

### 2.6. Influence of GM GT#4 Flaxseed Cake Feeding on Liver Ultrastructure in Mice

**Figure 6:**
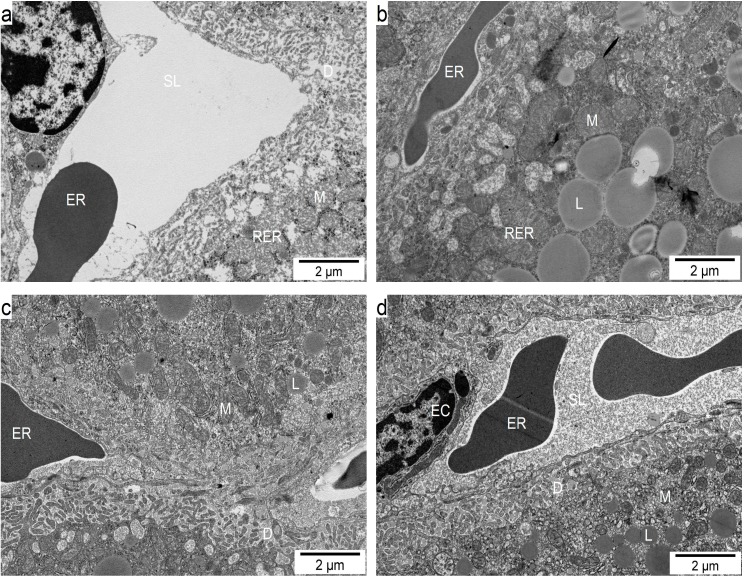
Transmission electron microscopy micrographs of the sinusoidal region of liver of mice fed the diets: (**a**) SD; (**b**) HFD; (**c**) Linola; and (**d**) GT#4. Mitochondrium (M), rough endoplasmic reticulum (RER), sinusoidal lumen (SL), space of Disse (D), endothelial cell (EC), erythrocyte (ER), lipid droplet (L), endothelial fenestrae (asterisk).

To further evaluate the potential liver protective effect of GM GT#4 flaxseed cake-based diet, ultrastructural examination was carried out by transmission electron microscopy (TEM) (Figure 6). The vascular surface of liver in the groups under GM GT#4 and non-GM flaxseed cakes, and also under SD (Figure 6a,c,d), was covered by microvilli regularly distributed in the Disse’s space, separating hepatocytes by the endothelial cells from the sinusoidal lumen. Liver from HFD-fed mice (Figure 6b) showed changes in the hepatocyte cell membrane directed to the Disse’s space where irregular decomposition of microvilli was observed. The structure, size and shape of hepatocyte mitochondria, and morphology of endoplasmic reticulum of feeding groups appear normal, even near cell membrane surface. In the hepatocytes of mice that administered HFD control, the gathering and excess lipid droplets of different size were observed. In the liver of mice that were fed GT#4 and non-GM flaxseed cakes, a specific decrease in the deposition of lipids was noted. Vacuolisation of some submembrane cytoplasmic regions was noted in some samples from different feeding groups. These results clearly indicate that diet supplementation with seed cake of GM GT#4 flax and the non-GM isogenic could have hepatoprotective action. In agreement with the findings from H&E staining and lipid assay, addition of GM GT#4 flaxseed cake to the diet improved steatosis when comparing to HFD-fed animals.

Male C57BL/6J mice that administered a Western diet (41% calories from fat, 43% calories from carbohydrate) for 6 months developed steatohepatitis and fibrosis. Feeding the Western diet also resulted in sinusoidal endothelium thickening, early capillarization and sinusoidal endothelial cell defenestration, and subendothelial basal lamina formation [70]. Defenestration and thickened endothelium could diminish the transfer of substrates between sinusoid and hepatocyte, especially lipoproteins [71]. The sinusoidal endothelium of liver is sensitive to oxidative stress. Its structure may be influenced by the dietary load quantity. Alterations in sinusoidal endothelium could have negative effects on liver function [72]. Defenestrated sinusoidal endothelium and subendothelial basement membrane presence characterize cirrhosis. Data from animal models indicate that defenestration precedes fibrosis that can lead to cirrhosis. Male Wistar rats that were given HFD (containing 10% hog fat, 2.5% cholesterol, 1% cholate, 10% sucrose) for 5 weeks and were administered additionally total flavonoids (mainly flavones and flavonols, including quercetin and kaempferol) from fruit of Rosa laevigata Michx (160 mg/kg) for 11 weeks had reduced microvesicular fatty changes and reversed mitochondrial injury induced by HFD [73]. Lipid accumulation in hepatocytes leads to dysfunction of mitochondria and oxidative stress.

## 3. Experimental Section

### 3.1. Plant Material and Transformation

Non-GM flax (*Linum usitatissimum* L. cv. Linola 947) seeds were received from the Flax and Hemp Collection of the Institute of Natural Fibers and Medicinal Plants (Poznan, Poland). GM GT#4 flax line was obtained in the Department of Genetic Biochemistry of the University of Wrocław (Wrocław, Poland). The GM line overexpresses *Solanum sogarandinum* cold-induced glucosyl transferase (SsGT1). The GT#4 flax line exhibits increased contents of kaempferol, quercetin glycosides, total anthocyanins, proanthocyanins, SDG, ferulic acid, *p*-coumaric acid relative to the control seeds. The genetic engineering techniques, selection methods, other procedures used for the production of the GM GT#4 flax line, as well as the genetic characterization and other compositional details can be found elsewhere [31,74]. The GM GT#4 and non-GM flax plants intended for our experiment were grown in a field in the vicinity of Wroclaw (trial No. 26, AM-13; decision of Polish Environment Ministry No. 36/2011 dated 29 September 2011). The fifth generation GM seeds that were harvested after 4 months of flax cultivation, in August 2012, were utilized.

### 3.2. Nutritional Study and Preparation of Animal Material

To assess the influence of flaxseed cake administration in a diet on the development of inflammation and to examine food safety aspects, a 96-day nutritional study was conducted on male mice which were derived from outbred stock by four inbred strains (A/St, BN/a, BALB/c and C57BL/6Jn) crossing. The experimental procedures were approved by the local ethics committee (Resolution No. 65/2010 of the III Local ethics committee on animal experiments in Warsaw dated 27 October 2010; pursuant to legal acts [75,76]). Mice, 30.1 g average initial body weight, were divided into four groups (*n* = 20) standardized in terms of their body weight, placed in growth cages (21 °C, 12 h/12 h, 40% humidity) and fed *ad libitum* one of the isoprotein diets: (1) standard diet (SD); (2) high-fat diet rich in pork lard (HFD); diet supplemented with 30% of seed cake of (3) the non-GM Linola (Linola) flax or (4) the GM GT#4 (GT#4) flax. All of the diets had been previously prepared in “Morawski” Feed Production Plant (Kcynia, Poland) and SD covered nutritional requirements of animals [77]. Diet composition and nutritional value which was determined according to the procedures of the Association of Official Analytical Chemists (AOAC) [78] were presented in Table 1. The access to water was free. Body weight gain of mice and food intake were recorded on a weekly basis. After an overnight fast, mice were anaesthetized by overdosing isoflurane (Aerrane, Baxter, Deerfield, IL, USA). Blood was collected from the eye in tubes intended for serum separation. After clotting blood samples were centrifuged (10 min, 3000 rpm), serum was aliquoted and stored (−25 °C). Samples of liver tissue (right lobe) were immediately frozen in liquid nitrogen and stored at −80 °C, put into formalin (4%) for light microscopic analysis or into 2.5% glutaraldehyde in phosphate buffer pH 7.2 for transmission electron microscopic examinations.

**Table 1:** Composition of diets and their nutritional value.

Component	Experimental Diet			
	SD	HFD	Linola	GT#4
Flaxseed cake (%)	-	-	30.0	30.0
Nutritional value (% dry matter)				
Crude protein	20.0	20.1	19.7	19.5
Crude fat	2.8	12.1	11.6	11.9
Crude fiber	4.0	3.8	4.9	4.6
Gross energy (kcal)	465	515	512	514
Gross energy from crude fat (kcal/100 g)	27	115	110	113

### 3.3. Total Antioxidant Status

Serum total antioxidant status (TAS) was recorded using commercial kit (Randox, Crumlin, UK). ABTS^®^ (2.2'-Azino-di-[3-ethylbenztiazoline sulphonate]) was incubated with a peroxidase (metmyoglobin) and H_2_O_2_ in order to ABTS^®^*^+^ production. The suppression of the color intensity by antioxidants was determined spectrophotometrically (600 nm) applying microplate reader (Infinite M200, Tecan, Männedorf, Switzerland).

### 3.4. Lipid Peroxidation

Lipid peroxides, major indicators of oxidative stress, were determined in serum as thiobarbituric acid reactive substances (TBARS) applying Uchiyama and Mihara technique [79]. Malondialdehyde (MDA), the product of the process of lipid peroxidation, forms with thiobarbituric acid (TBA) an 1:2 adduct that concentration was recorded spectrophotometrically. To standard curve construction was used MDA standard and TBARS were expressed as their equivalents. Absorbance was measured at 532 nm (microplate reader).

### 3.5. Serum Lipid Profile

Indices of serum lipid profile: concentration of total cholesterol (TC), high-density lipoprotein (HDL) cholesterol and triglycerides (TG) were determined by spectrometric method, applying a VITROS^®^ DT60 II Analyzer (Johnson & Johnson Clinical Diagnostics, New Brunswick, NJ, USA). TC/HDL ratio was calculated.

### 3.6. Inflammatory State Markers

To examine the concentration of inflammation indicators in liver, tissue samples of this organ had been homogenized in cold 0.02 M phosphate-buffered saline (PBS) using a metal homogenizer (Polytron^®^ PT2100, Kinematica AG, Lucerne, Switzerland), centrifuged (30 min, 4 °C, 15,000 rpm) and the supernatant was collected. Protein concentration of the samples was determined using the Peterson’s modification of the method of Lowry applying a commercial kit (Sigma, St. Louis, MO, USA). Levels of inflammatory state markers in liver homogenates were assessed using enzyme-linked immunosorbent assay—ELISA (Wuhan EiAab Science Co., Ltd., Wuhan, China). The absorbance was measured using a microplate reader (450 nm).

### 3.7. Hepatic Histological Evaluation

The liver fragments fixed with formalin were embedded in paraffin and sliced for 5 µm thickness according to the routine procedure. The obtained sections were stained with hematoxylin and eosin (H&E). Then the sections were photographed under a light microscope Leica DM750 (Leica Microsystems Ltd., Heerbrugg, Switzerland), at 20× magnification.

### 3.8. Transmission Electron Microscopic Examination of Liver

Liver pieces that were prefixed with glutaraldehyde were post-fixed with 1% osmium tetroxide and embedded in Epon for transmission electron microscopy (TEM). Ultrathin sections were stained with uranyl acetate and lead citrate, and examined under a JEOL JEM-1220 electron microscope (JEOL Ltd., Tokyo, Japan).

### 3.9. Statistical Analysis

Results are expressed as mean ± SEM. One-way analysis of variance (ANOVA) was applied with means compared using the Least Significant Difference (LSD) correction. A difference of *p* < 0.05 between means was considered to be significant. Statgraphics Centurion software (StatPoint Technologies, Inc., Warrenton, VA, USA) was used.

## 4. Conclusions

In this work, the impact of diet supplemented with GM GT#4 flaxseed cake, enriched with SDG, ferulic and *p*-coumaric acids, kaempferol, quercetin and their glycosyl derivatives, on inflammation development in mice model was studied. Feeding a diet that contained GM GT#4 flaxseed cake statistically significantly improved the total antioxidant status in mice serum and this index did not differ from Linola. GM and isogenic flaxseed cake administration positively influenced on TBARS. Further, the diet supplemented with GM and non-GM flaxseed cake improved lipid profile in mice serum. Also beneficial was the reduction in the level of pro-inflammatory TNF-α agent not only in the GT#4 flaxseed cake group but also in the isogenic one. Feeding GT#4 flaxseed cake reduced CRP concentration which did not differ from non-GM. Consumption of seed cake of both lines improved liver structure. The interesting observation was that administration of GM and isogenic flaxseed cake lowered mice body weight gain, which is a good prognosis for obesity prophylaxis. According to the results obtained in the present study, GM seed cake, as well as isogenic seed cake, may have a beneficial role as diet supplements in promoting health and wellness.
